# Time Is a Critical Factor When Evaluating Oligonucleotide Therapeutics in hERG Assays

**DOI:** 10.1089/nat.2022.0043

**Published:** 2023-03-30

**Authors:** Yusheng Qu, Robert Kirby, Richard Davies, Ayesha Jinat, Stefano Stabilini, Bin Wu, Longchuan Yu, BaoXi Gao, Hugo M. Vargas

**Affiliations:** ^1^ Amgen Research, Translational Safety and Bioanalytical Sciences, Amgen, Inc., Thousand Oaks, California, USA.; ^2^ Metrion Biosciences Ltd, Granta Center, Cambridge, United Kingdom.; ^3^ Hybrid Modality Engineering, Amgen, Inc., Thousand Oaks, California, USA.; ^4^ Cardiometabolic Disorders, Amgen, Inc., Thousand Oaks, California, USA.

**Keywords:** siRNA, oligonucleotide, hERG, PCR, automated electrophysiology

## Abstract

In accord with International Conference on Harmonization S7B guidelines, an *in vitro* human ether-a-go-go-related gene (hERG) assay is one component of an integrated risk assessment for delayed ventricular repolarization. Function of hERG could be affected by direct (acute) mechanisms, or by indirect (chronic) mechanisms. Some approved oligonucleotide therapeutics had submitted hERG data to regulatory agents, which were all collected with the same protocol used for small-molecule testing (incubation time <20 min; acute), however, oligonucleotides have unique mechanisms and time courses of action (indirect). To reframe the hERG testing strategy for silencing RNA (siRNA), an investigation was performed to assess the time course for siRNA-mediated inhibition of hERG function and gene expression. Commercially available siRNAs of hERG were evaluated in a stable hERG-expressed cell line by whole-cell voltage clamp using automated electrophysiology and polymerase chain reaction. In the acute hERG study, no effects were observed after treatment with 100 nM siRNA for 20 min. The chronic effects of 100 nM siRNAs on hERG function were evaluated and recorded over 8–48 h following transfection. At 8 h there was no significant effect, whereas 77% reduction was observed at 48 h. Measurement of hERG mRNA levels demonstrated a 79% and 93% decrease of hERG mRNA at 8 and 48 h, respectively, consistent with inhibition of hERG transcription. The results indicate that an anti-hERG siRNA requires a long exposure time (48 h) in the hERG assay to produce a maximal reduction in hERG current; short exposures (20 min–8 h) had no effect. These findings imply that off-target profiling of novel oligonucleotides could benefit from using hERG protocol with long incubation times to de-risk potential off-target (indirect) effects on the hERG channel. This hERG assay modification may be important to consider if the findings are used to support an integrated nonclinical–clinical risk assessment for QTc (the duration of the QT interval adjusted for heart rate) prolongation.

## Introduction

Successful drug development with oligonucleotide has now matured to the position of therapeutic usage for multiple indications [1,2]. Small interfering RNA, sometimes known as short interfering RNA or silencing RNA (siRNA), is a class of double-stranded noncoding RNA molecules, typically 20–27 base pairs in length, and operating within the RNA interference pathway [3,4]. Targeted gene suppression by siRNA and ultimately, regulating target protein biosynthesis, has become an exciting area for developing therapeutics of previously undruggable targets [5] and there are ∼10 siRNA molecules either approved as marketed drugs or in the late-stage clinical development [2]. Currently, there is no specific regulatory guidance for nonclinical safety pharmacology assessment of oligonucleotide-based therapeutics. The industry practice is following a hybrid of the small-molecule (SM) International Conference on Harmonization (ICH) guidance (S7A and S7B) and biologics guidance (ICH S6). In addition, the recommendations of the Oligonucleotide Safety Working Group (OSWG, eg, Berman *et al.* [6], Marlowe *et al.* [7]) are used to help design preclinical studies for this modality.

The human ether-a-go-go-related gene (hERG; KCNH2-human gene symbol) encodes a potassium channel responsible for the rapidly activating delayed rectifier K^+^ current (IKr), which mediates the late repolarization phase of ventricular action potentials [8]. The pharmacological susceptibility of hERG to blockade by a variety of structurally diverse drugs underlies the drug-induced form of acquired long-QT syndrome (aLQTs), which could lead to Torsades de Pointes (*TdP*), a life-threatening disturbance of heart's rhythm [9,10]. The evidence for hERG in aLQTs and arrhythmias is sufficiently strong that the ICH S7B guideline describes an *in vitro* hERG study as a component of the strategy for an integrated risk assessment of QT prolongation when developing a new chemical entity (NCE). There is high confidence in the predictive value of hERG assay findings by both the pharmaceutical industry and regulatory community to characterize NCE for QTc (the duration of the QT interval adjusted for heart rate) prolongation and *TdP* risk [11,12].

Function of hERG could be affected by direct and acute mechanisms, such as blockade of SM and peptides. It could also be inhibited by indirect and chronic mechanisms, such as gene silencing and protein trafficking interruption. Direct inhibition occurs when there is a physical interaction between an agent and hERG channel. Majority of SM inhibitors of hERG, including dofetilide [13], interact with two hydrophobic residues, Phe 652 and Tyr 656, in the S6 domain on the cytosolic side of the ion selectivity filter [8,14]. In addition, peptide inhibitors of hERG, such as BeKm-1 [15], have been shown to bind to the extracellular region on the N-terminal side of the re-entrant pore loop spanning between transmembrane domains S5 and S6 [15]. On the other hand, hERG function could also be affected by indirect and chronic mechanisms, such as gene silencing [16] and trafficking inhibition [17,18]. Trafficking interrupters, such as pentamidine and probucol, have been shown to downregulate hERG function [17,18] in the timeframe of 16–48 h.

Specific siRNA designed for hERG has been used to knockdown hERG function [16] by transfecting the myocytes for 48 h. Consistently, it is well established that siRNA-mediated gene silencing takes ∼48–96 h for inhibition of protein function [19,20], indicating that an acute application in hERG testing of oligonucleotides would miss inhibitory effects due to gene silencing.

What is the appropriate protocol to detect hERG effects with novel modalities, like siRNA, which have unique intracellular mechanisms and time courses of action? The electrophysiological protocols for testing direct effects of SM or peptide on hERG channel are typically in an acute assay format, where the test article is applied for <20 min.

To help frame the strategy of hERG assay for siRNA, investigation was performed to assess the time course for siRNA-mediated inhibition of hERG function and gene expression.

## Methods

### Cell culture

Chinese Hamster Ovary (CHO) cells stably expressing hERG cDNA (CHO-hERG) were cultured in a media of Ham's F-12 Nutrient Mix, GlutaMAX™ Supplement (Catalog number: 31765027) with 10% fetal bovine serum, 100 μg/mL Geneticin, and 100 μg/mL Hygromycin.

### hERG siRNA tool molecules (KCNH2 siRNA): the positive control

A tool siRNA (KCNH2 siRNA), which is a mixture of four siRNA molecules, was used in this study to understand the concentration- and time-dependent relationships between test article treatment and impact on hERG functional responses. The KCNH2 siRNA was used to specifically knockdown hERG expression and served as the positive control. This molecule had sense strand sequences shown in the following that target the nucleotides of c-linker regions as well as the S5 side of the P-loop of hERG.

*Homo sapiens* KCNH2 transcript variant 1 mRNA (NM_000238.4)

- siRNA 1 targeting location: 2015–2033: CGCGGAAGCUGGAUCGCUA; c-linker region
- siRNA 2 targeting location: 1844–1862: ACGAGGAGGUGGUCAGCCA; between S5P and P
- siRNA 3 targeting location: 2175–2193: GGGCGACCAGAUAGGCAAA: c-linker region
- siRNA 4 targeting location: 2140–2158: CACAUGGACUCACGCAUCG: c-linker region

Both KCNH2 siRNA and nontargeting control siRNA were ordered from Horizon Discovery (Dharmacon). (Catalog ID: L-006233-00-0050 for KCNH2 siRNA; Catalog ID: D-001810-01-50 for nontargeting control siRNA). A nontargeting control siRNA with the sequence of UGGUUUACAUGUCGACUAA was used as a negative control to exclude nonspecific effects of siRNA, which will be described as a negative siRNA for simpler narrations

To understand if siRNA transfection affected cell viability, Nexcelom T4 cell counter (Nexcelom Bioscience, MA) was used to measure viability. The Nexcelom T4 cell counter utilizes bright-field imaging and pattern-recognition software to identify and count individual cells. Viability was assessed with Trypan Blue, which is a dye exclusion method that utilizes membrane integrity to identify dead cells. The dye is unable to penetrate healthy cells, so they remain unstained. Dead cells have a compromised cell membrane that is permeable to the Trypan Blue dye. Dead cells are stained blue and display as dark cells in the Cellometer software with bright-field imaging. The cell concentration and % viability for cells stained with Trypan Blue were automatically calculated and reported.

### Electrophysiological protocol of the hERG assay

The hERG channel experiments were performed using automated patch clamp electrophysiology with the QPatch 48 system (Sophion, Denmark), where hERG currents were recorded in conventional whole-cell voltage clamp with single-hole QPlates. The following recording solutions were used (in mM): extracellular, NaCl, 140; KCl, 2; CaCl_2_, 2; MgCl_2_, 1; HEPES, 10; Glucose, 5; pH = 7.40 adjusted with NaOH; intracellular, KF, 60; KCl, 70; EGTA, 5; MgATP, 5; HEPES, 10; pH = 7.20 with KOH. A standardized voltage protocol was used to elicit ionic current through the hERG potassium channel.

From a holding potential of −80 mV, the cell was initially depolarized to −50 mV (500 ms) to allow measurement of instantaneous leak current (without hERG activation) before a further depolarization to +30 mV (2 s) to activate/inactivate the channel resulting in an outward current. The cell membrane was repolarized from +30 to −50 mV (2 s) to relieve inactivation, during which a characteristic resurgent hERG tail current was observed, which slowly deactivated, before finally returning to the holding potential. The command voltage waveform was repeatedly applied every 10 s. Peak outward tail current amplitude elicited at −50 mV was analyzed as hERG channel function. Electrophysiology experiments were performed at room temperature (18–22°C).

### Acute hERG assay

A single high concentration of 100 nM KCNH2 siRNA was initially used to enable maximal gene knockdown. The siRNAs were prepared to a 20 μM stock concentration using sterile ultrapure (nuclease free) water. Testing solutions were prepared as a 1:200 dilution of the 20 μM stock into extracellular recording solution. Vehicle control was prepared by adjusting the extracellular recording solution accordingly using water. The siRNA molecules were applied as 12 solution exchanges over a 20-min period. Furthermore, a standard reference compound, verapamil, was evaluated at 0.3, 1, 3, and 10 μM using the same electrophysiology protocol to confirm assay sensitivity. Four cumulative concentrations of verapamil were applied for 5 min at each concentration.

### Transfection of siRNA in CHO-hERG cells

DharmaFECT 4 (Cat No: T-2004-03) transfection reagent was selected for transfection into CHO-hERG as this is the proprietary transfection reagent and method provided by the manufacturer (Horizon Discovery) of the siRNA molecules.

The maximal assessment time point of 48 h was selected because DharmaFECT protocol recommended that 48–96 h was required to observe siRNA effects on protein production.

CHO-hERG cells were seeded at 0.1, 0.3, and 0.5 × 10^6^ cells for 24–48 h into T-75 flasks, from which those with the optimal confluency were selected for transfection (∼30% coverage).

### Effects of siRNA transfection on hERG currents

#### Time dependence

To understand the time-dependent effects of KCNH2 siRNA on hERG channel function, the effects of 100 nM siRNA, including KCNH2 siRNA and negative control siRNA on the functional expression of the hERG channel at 8, 16, 24, and 48 h posttransfection were investigated. At each time point, the study was composed of three experimental Groups, (1) Mock transfection; (2) negative siRNA; and (3) KCNH2 siRNA.

#### Concentration dependence

The effects of 10, 30, and 100 nM KCNH2 siRNA on the hERG current level 48 h after transfection into hERG CHO cells were compared with time-matched negative siRNAs and mock transfection.

#### Measurement of hERG currents after siRNA transfection

The electrophysiology protocol and solutions were the same as in the acute hERG study. When a quality recording was established, cells were washed for 2 min, followed by applying extracellular solution for 5 min. The current amplitudes were measured at the end of 5 min. A supramaximal concentration of a selective hERG channel blocker (1 μM E-4031) was added at the end of each experiment for 1 min to determine the amplitude of hERG currents. The E-4031-sensitive currents were taken as the hERG currents. A minimum of 20 cells were recorded for each experimental group.

#### Measurement of KCNH2 mRNA after siRNA transfection

Cells were harvested with QIAzol^®^ Lysis Reagent, and QIAGEN RNeasy Plus 96 Kit was used for RNA purification. The cell lysates were homogenized for 70 s at 6500 rpm with MagNA Lyser Green Bead tubes in the MagnaLyser (Roche Diagnostics). RNase-Free DNase Set (QIAGEN Cat No. 79254) was used to remove genomic DNA contamination on column.

After RNA purification, reverse transcription samples were generated with Invitrogen™ SuperScript™ III First-Strand Synthesis SuperMix for qRT-polymerase chain reaction (PCR).

KCNH2 expression was evaluated by droplet digital PCR (ddPCR) and the concentrations of mRNA was recorded and analyzed with NanoDrop 8000. Hprt1 was measured as a housekeeping gene in CHO cells and used for normalization of KCNH2 expression levels. Duplicate samples from two independent experiments were analyzed at each condition.

### Data analysis

Data were presented as mean and standard error of the mean (SE). Curve fitting for time- and concentration-dependent effects were performed in either GraphPad Prism (GraphPad Software LLC) or in SigmaPlot Software (SYSTAT Software, Inc.) using Nonlinear Regression—Dynamic Fitting of Exponential Rise to Maximum with the equation f = a*(1-exp(−b*x)), where a = minimal effect, b = slope, x = drug concentration, and f = effect at drug concentration of x.

Statistical analysis was performed in GraphPad Prism (GraphPad Software, LLC) with one-way analysis of variance (ANOVA) followed by a Tukey's multiple comparisons test. *P* < 0.05 was considered as significant.

## Results

### The KCNH2 siRNA (positive control) had “no effect” in the acute hERG assay

**FIG. 1.**
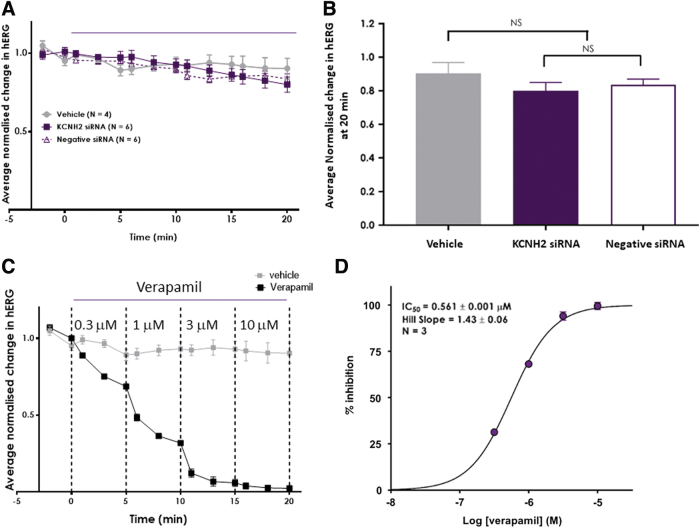
KCNH2 siRNA did not inhibit hERG currents during 20 min application. **(A)** hERG currents normalized by baseline were averaged (mean ± SE) under vehicle control (*solid gray symbol*), 100 nM KCNH2 siRNA (*solid purple symbol*), and 100 nM negative siRNA (*open purple symbol*). Liquid exchanges were performed twice in baseline, 3 times every 5 min, and a total of 12 times in 20 min. The current amplitudes were analyzed at the end of an addition before the next addition. **(B)** hERG currents normalized by baseline (mean ± SE) at the end of 20-min application of vehicle (*solid gray*), 100 nM KCNH2 siRNA (*solid purple*), and 100 nM negative siRNA (*open purple*). **(C)** Time-dependent effects of verapamil (0.3, 1, 3, and 10 μM) on hERG currents. The hERG currents normalized by baseline were averaged (mean ± SE) under vehicle control (*gray symbol*) and four concentrations of verapamil (*black symbol*). Verapamil application was performed three times in a duration of 5 min at each concentration. **(D)** Concentration-dependent inhibition of hERG by verapamil. NS, not statistically significant; SE, standard error of the mean; siRNA, silencing RNA.

In Fig. 1A, the effects of vehicle, KCNH2 siRNA, and negative siRNAs on hERG current were measured at the end of each liquid addition period and normalized to baseline (time at −2 and 0 min). The current changes under three experimental conditions were similar to each other during the 20 min application.

Mean and SE of normalized hERG currents from Fig. 1A were derived at the end of the last addition period (20 min, Fig. 1B). Statistical analysis showed that there was no statistical difference observed between the effect measured for 100 nM negative siRNA when compared with the effect by 100 nM KCNH2 siRNA. Furthermore, the effect of both siRNAs was not statistically different when compared with the vehicle group (Fig. 1B).

On the other hand, verapamil (positive control) inhibited hERG currents in a time-dependent (Fig. 1C) and concentration-dependent manner (Fig. 1C, D) with a mean IC_50_ of 0.561 μM. The potency of verapamil in the present study was consistent with historical data (IC_50_: 0.587 μM, with a 95% confidence interval of 0.553–0.623, *n* = 32 studies at Metrion Biosciences) and prior literature (eg, Crumb *et al.* [21]). The potency and time dependence of verapamil's effect confirmed assay sensitivity.

### Time-dependent effects of KCNH2 siRNA on hERG currents

To understand if transfection of siRNA would affect cell health and recordings, cell viability was evaluated after transfection of siRNA for 48 h, which was 98.6% after mock transfection, 80.4% after 100 nM nontargeting siRNA transfection, and 85.2% after 100 nM KCNH2 siRNA transfection. The decrease of cell viability did not affect the recordings because the number of viable cells were sufficient at each condition.

**FIG. 2.**
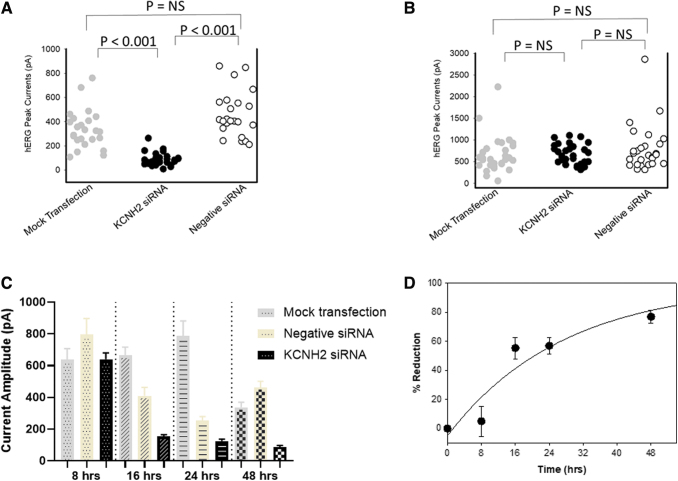
Time-dependent inhibition of hERG currents by siRNA at 8, 16, 24, and 48 h. **(A, B)** Current amplitude recorded from each cell in one experiment was analyzed and shown under each condition. Mock transfection (*gray symbol*), KCNH2 siRNA transfection (*black symbol*), negative siRNA transfections (*open symbol*). **(A)** The amplitude of hERG currents was significantly decreased after 48 h transfection of KCNH2 siRNA. **(B)** The amplitude of hERG currents was not significantly decreased after 8 h transfection of KCNH2 siRNA. **(C)** Average hERG current amplitude (mean ± SE) in the same experiment after mock transfection, negative, and KCNH2 siRNA transfection as labeled. **(D)** Mean percent reduction of hERG currents (*n* = 2 experiments) by KCNH2 siRNA compared with negative siRNA at different time points after transfection. The percent inhibition was derived by the following formula (1−[mean hERG current amplitude in KCNH2 siRNA-transfected cells]/[mean hERG current amplitude in negative siRNA-transfected cells]).

To evaluate the time-dependent effects on hERG function by KCNH2 siRNA transfection, two independent experiments were performed, and Fig. 2A–C shows one example. Figure 2D shows the average of two experiments. To investigate if a duration of 48 h is long enough for KCNH2 siRNA to inhibit hERG function, the effects of 100 nM KCNH2 siRNA and negative siRNA on hERG currents were evaluated and compared with a time-matched mock transfection after 48 h (Fig. 2A). In mock transfected cells, hERG current amplitude was 337.0 ± 32.4 pA (*n* = 24 cells); in KCNH2 siRNA-transfected cells, hERG current amplitude was 86.4 ± 10.5 pA (*n* = 27 cells); in negative siRNA-transfected cells, hERG current amplitude was 462.1 ± 39.5 pA (*n* = 23 cells). There was a significant (*P* < 0.001) reduction in the mean current amplitude from KCNH2 siRNA-transfected cells when compared with mock and the negative siRNA-transfected cells. When comparing the current amplitudes in the mock or negative siRNA-transfected cells, there were no statistical differences.

To understand the time-dependent effects of KCNH2 siRNA, three more time points were selected, which were 8, 16, and 24 h posttransfection.

Figure 2B showed the individual cell hERG current amplitude recorded at 8 h posttransfection. In mock transfected cells, hERG current amplitude was 639.4 ± 67.5 pA (*n* = 34 cells); in KCNH2 siRNA-transfected cells, hERG current amplitude was 640.4 ± 40.2 pA (*n* = 36 cells); in negative siRNA-transfected cells, hERG current amplitude was 795.3 ± 101.8 pA (*n* = 27 cells). There was no significant difference in the mean current amplitude from KCNH2 siRNA-transfected cells when compared with mock and negative control siRNA. In addition, there was no significant difference in the current distributions recorded between negative siRNAs and mock transfection.

Figure 2C shows the summarized data recorded at 8, 16, 24, and 48 h. At 16 and 24 h posttransfection, there was a significant (*P* < 0.05) reduction in the mean current amplitude of the negative siRNA-transfected cells when compared with mock control. At both time points, when comparing the KCNH2 siRNA-transfected cells with the negative siRNA-transfected cells, the average hERG current amplitude was reduced.

The average time-dependent reduction of hERG function was derived in Fig. 2D with the following formula, (1−[mean hERG current amplitude in KCNH2 siRNA-transfected cells]/[mean hERG current amplitude in negative siRNA-transfected cells]) for each experiment.

### Concentration-dependent effects of siRNA

**FIG.3.**
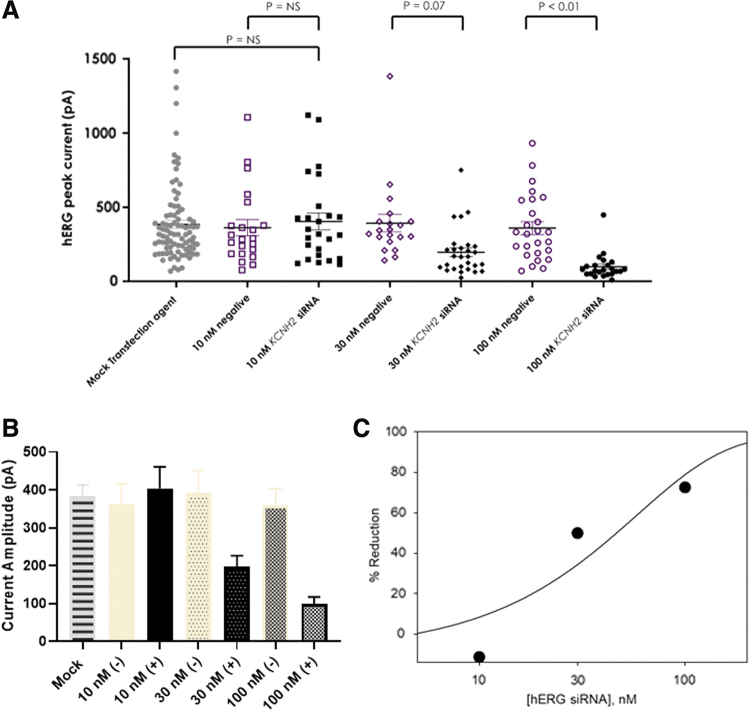
Concentration-dependent inhibition of hERG current by siRNA after 48 h of transfection. **(A)** Current amplitude recorded from each cell shown under each condition as labeled. **(B)** Average current amplitude (mean ± SE) in each transfection condition as labeled. (+): KCNH2 siRNA, (−): negative siRNA. **(C)** Percent reduction of mean hERG currents by 10, 30, and 100 nM positive hERG siRNA compared with hERG currents after negative siRNA transfection.

To understand the concentration-dependent effects of siRNA, three concentrations of siRNA, 10, 30, and 100 nM, were transfected and hERG function was evaluated at 48 h posttransfection. Figure 3A shows the hERG current amplitude recorded from each cell and Fig. 3B shows the summarized data at each testing condition. Consistent with previous observations at 48 h posttransfection (Fig. 2A), the negative siRNA had no significant impact on the current amplitude compared with mock transfection. There was no statistically significant effect of HCNH2 siRNA at 10 nM when compared with the negative control. At 30 nM, there was a 50% mean reduction in the current, however, the effect was not statistically significant (*P* = 0.07). At 100 nM, a significant lower current level was recorded compared with the negative siRNA with a 72% reduction, consistent with previous observation described in Fig. 2A when the time course was investigated.

### Time-dependent effects of siRNA on hERG mRNA levels

**FIG. 4.**
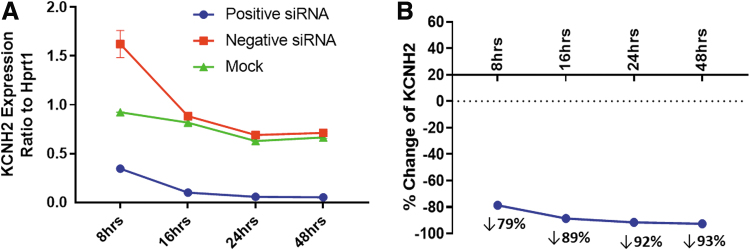
Time-dependent decrease of KCNH2 expression by siRNA. **(A)** KCNH2 expression levels were analyzed by digital droplet PCR and normalized by a housekeeping gene, Hprt1. Each time points were measured in duplicate samples in mock transfection, KCNH2 siRNA and negative siRNA transfection as labeled. **(B)** Percent change of KCNH2 expression was derived by comparing mean KCNH2 mRNA level in KCNH2 siRNA transfection with that in negative siRNA transfection. PCR, polymerase chain reaction.

To further investigate the mechanism of siRNA's effect on hERG function, the time-dependent effects of KCNH2 siRNA on hERG expression at 8, 16, 24, and 48 h were evaluated with ddPCR. Figure 4A describes the KCNH2 mRNA levels normalized by Hprt1, a housekeeping gene in CHO cells. The HCNH2 mRNA levels were similar between mock and negative siRNA transfection starting at 16 h, however, they were elevated in negative siRNA-transfected cells at 8 h. Compared with mock and negative siRNA transfection, the mRNA levels were reduced at all four time points in the KCNH2 siRNA-transfected cells.

To illustrate the time-dependent effects, the percent changes in KCNH2 mRNA levels by KCNH2 siRNA transfection were normalized by the mRNA levels in negative siRNA transfection in Fig. 4B.

## Discussion and Conclusion

To our knowledge, the kinetics of hERG inhibition by a specific KCNH2 siRNA has not been evaluated previously in the *in vitro* hERG assay. When tested in an acute protocol designed for a direct-acting SM, the anti-hERG siRNA-positive control yielded a false-negative result. Even at 8 h posttransfection, the effect on hERG function was not detected although gene knockdown was apparent with PCR analysis. These observations suggest that 48 h are required to address whether a siRNA inhibits hERG function through a gene silencing mechanism. These findings are consistent with previous conclusions that a longer time window, for example, 48–96 h, is necessary for siRNA-mediated gene silencing [19,20].

Our study provides indisputable evidence that the hERG assay protocol appropriate for direct channel blockers is inadequate to detect an effect by siRNA because hERG protein downregulation by gene silencing requires longer time. The findings support the rational design of experimental protocols when examining the hERG liability by a novel oligonucleotide drug if an hERG study is suggested and highlight the need for best practice in safety pharmacology methodologies when evaluating oligonucleotide therapeutics.

### Oligonucleotides are different from SM mechanistically

Specific gene silencing by siRNA offers the potential to clarify gene function and modulate cellular mechanisms of disease [22], which recognize specific mRNA sequences by complementary Watson–Crick base paring [4,23]. Although the manufacture of oligonucleotides is a chemical process, like traditional SM drugs [24], the high degree of single-target specificity of siRNA [4,23] and low off-target risk are more aligned with large molecules (eg, monoclonal antibodies), which are designed to have high specificity for their target/epitope [25], and have little ability to directly block the hERG channel [26].

There are reports that siRNA can produce off-target effects. For example, siRNA may bind and repress mRNAs with partial sequence complementarity [27] and have the potential of silencing unintended targets to produce off-target effects [28,29]. SM therapeutics produce off-target effects by physically and reversibly binding to the off-target protein [8,30], while siRNAs influence off-target function by knocking down gene expression, an indirect mechanism [29]. The inhibitory effect on protein function by SM occurs promptly after binding [31], whereas the pharmacological effect on protein function following gene silencing by siRNA is downstream and takes hours or days to be observed [19,20]. The differences between a siRNA and a SM are depicted by comparing the effect of a KCNH2 siRNA with verapamil in the hERG assay. Verapamil's effect was detected within 5 min of drug application, but the effects of the siRNA took 48 h to manifest. These findings indicate that novel oligonucleotide therapeutics require longer incubation times to probe for indirect or chronic off-target effects in the hERG assay.

### Protein functional assessment is the ultimate test of siRNA effects

To understand the siRNA effects, the measurement of mRNA expression levels of the target gene is the first step. The following step is to examine the siRNA effects at the protein level. Ultimately, confirmation of the siRNA effects should be elucidated by analyzing the protein function. The mRNA levels may not always correlate with protein levels. For example, mRNA measurement can overestimate knockdown of genes whose protein products have long half-lives [20]. The hERG channel is a good example of a protein with a slow turnover rate of ≈11 h [32,33]. The studies reported here provide an excellent example for such a case. When the KCNH2 mRNA levels were measured with PCR, 79% reduction of hERG mRNA was achieved at 8 h after siRNA transfection, but there was only a 5% decrease in hERG currents, that is, the definitive measure of hERG function.

**FIG. 5.**
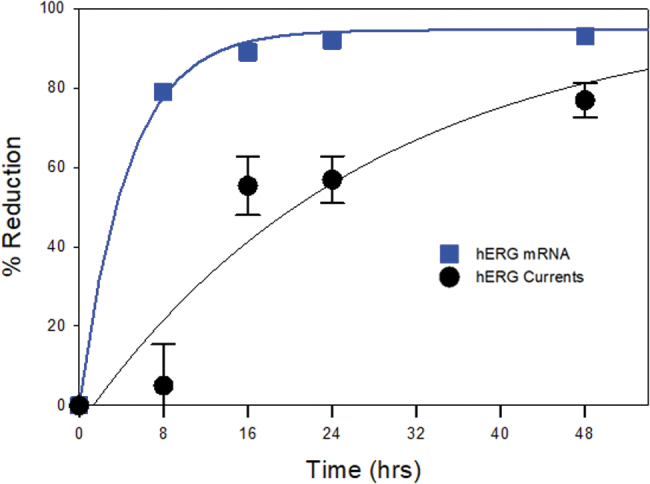
Knockdown of KCNH2 gene expression occurred before hERG functional inhibition. Overlay plots of percent change of KCNH2 expression and percent reduction of hERG currents as labeled. Percent change of KCNH2 expression was derived by comparing mean KCNH2 mRNA level in KCNH2 siRNA transfection with that in negative siRNA transfection. Mean percent reduction of hERG currents was from Fig. 2D.

This discrepancy of time course is illustrated in Fig. 5, which overlays the time course of reduction in KCNH2 mRNA levels with hERG current magnitude. The effects of KCNH2 siRNA on hERG function were clearly proceeded by a reduction in KCNH2 mRNA, consistent with the mechanism of gene silencing by siRNA [4,23] and the slow turnover rate of hERG [32]. Therefore, to understand the effects of a siRNA against a specific target, determination of mRNA levels of the target gene is not sufficient. Rather, it is necessary to assess protein levels and function to determine the optimal time point for assessing effects of gene knockdown.

### Limitations

There are several limitations in this study. The most critical one is the limited number of cells (20–38 cells per experimental condition) recorded at each condition. The variability of hERG current amplitude combined with the limited number of cells recorded may lower sensitivity for detecting a true effect. For example, 30 nM KCNH2 siRNA decreased hERG currents by ∼50%, but the effects were not statistically significant (*P* = 0.07). The second limitation is the inability to quantify the actual “free concentration” of siRNA molecules used in this study, given that oligonucleotides can exhibit significant binding to plasma proteins [34,35]. Although the hERG assay buffer is protein free, the medium for culture and transfection of hERG CHO cells contains 10% fetal bovine serum, which would bind siRNA during transfection [36]. In addition, the siRNA getting into the CHO cells would bind to intracellular proteins, therefore, the available free siRNA is uncertain, but likely to be less than the intended concentration.

Lastly, the variability of hERG current amplitude recorded at different time points and the mechanism of hERG current reduction by negative siRNA at 16 and 24 h posttransfection are unknown. The lack of KCNH2 mRNA decrease by negative siRNA at the same time points suggests that the negative siRNA does not silence the KCNH2 gene. To exclude nonspecific effects of siRNA, it is important to include a negative siRNA in the study design.

### Proposed strategy of assessing siRNA in a hERG assay

Due to the high degree of single-target specificity of siRNA [4,23], its nonspecific off-target potential at hERG is expected to be low. In addition, for many siRNAs in clinical development, the heart is not a target organ [2], for example, GalNAc-conjugated antisense oligos display highly specific delivery and internalization by hepatocytes primarily, but not other organs [37]. Thus, oligonucleotides that are engineered to deliver their payload to specific organs would have minimal exposure in the heart and low risk for interaction with the intracellular machinery of cardiomyocytes, including hERG channel synthesis pathways. Based on this line of thinking, the OSWG has proposed that a conventional *in vitro* hERG assay has little value for off-target risk assessment [6]. Furthermore, a recent draft guidance on nonclinical safety assessment of antisense oligonucleotides suggests that a hERG assay is generally not warranted.

If there are concerns that an oligonucleotide could penetrate cardiac myocytes, and/or an identified potential to interfere with any gene expression related to the IKr channel, the *in vitro* hERG investigation should include application times of ≥48 h posttransfection. This time frame is supported by results presented here and prior reports with oligonucleotides [19,20].

## Conclusion

Studies performed in this report clearly demonstrate that duration of siRNA application is an important factor in assessing the potential for hERG-mediated risk. The time course study indicated that when a KCNH2 siRNA (positive control) was applied for 20 min or 8 h, no inhibitory effect on hERG function was detected. These results suggest that a protocol with short application time for detecting direct hERG block yields a false-negative hERG risk. If a novel oligonucleotide therapeutic needs to be tested in a hERG assay, longer incubation times to probe for indirect off-target effects should be considered.
